# Increasing the Understanding and Demonstration of Appropriate Affection in Children with Asperger Syndrome: A Pilot Trial

**DOI:** 10.1155/2011/214317

**Published:** 2011-11-24

**Authors:** Kate Sofronoff, Johann Eloff, Jeanie Sheffield, Tony Attwood

**Affiliations:** ^1^School of Psychology, The University of Queensland, St Lucia, Brisbane, QLD 4072, Australia; ^2^School of Psychology, Griffith University, Mt Gravatt, Brisbane, QLD 4122, Australia

## Abstract

The study was conducted to examine relationships between affectionate behavior in children with Asperger syndrome and variables likely to influence its expression (e.g., tactile sensitivity, social ability). It also evaluated the impact of a cognitive behavioral intervention that aimed to improve a child's understanding and expression of affection. Twenty-one children, aged 7 to 12 years, participated in the trial. The results showed significant correlations between measures of affection and tactile sensitivity and social ability. After attending the 5-week program, parents identified significant increases in the appropriateness of children's affectionate behavior both towards immediate family and people outside the immediate family, despite reporting no significant changes in their child's general difficulties with affectionate behavior. There was a significant improvement in children's understanding of the purpose of affection. The findings are discussed as well as the limitations of the study.

## 1. Introduction

Affectionate communication is a form of social behavior that individuals with Asperger syndrome (AS) find problematic [1]. Affectionate communication comprises those actions through which humans express feelings of positive regard such as liking, love, closeness, care, and gratitude to one another by means of verbal, nonverbal, and supportive forms of communication [2–4]. The capacity to express and receive affection is considered a fundamental human need by social scientists such as Rotter et al. [5] and is considered vital for the formation, maintenance, and quality of personal relationships [2].

Children with AS have great difficulty in understanding affection and expressing appropriate levels of affection [1]. The clinical presentation of AS has many features that overlap with the personality trait alexithymia [6], in which the individual has an impaired ability to recognise, process, and communicate their emotional state. Many individuals with AS demonstrate an impaired capacity to recognise and communicate emotion-related information about themselves and others [7]. According to Attwood [1, 8], those with AS are particularly likely to misread the intentions of others and may inadvertently engage in behaviors that are socially unacceptable or inappropriate in terms of touch, personal space, greetings, and gestures of affection that can lead to embarrassment in others. 

Parents of a child with AS face an increased number of stressors and report greater stress compared with parents of a typical child [9] or a child with another developmental disorder [10]. They also report distress associated with their child's infrequent expressions of affection [1, 11]. For example, a mother complained that her adolescent son with AS did not express enough affection, to which he replied that he said that he loved her when he was six and was puzzled as to why he should repeat this phrase [8]. The absence of affection in close relationships is correlated with loneliness [12] and depression [13] and is an indicator of relational deterioration [2]. Hence, the difficulties associated with emotional reciprocity in AS are likely to reduce a parent's experience of reinforcement in their parenting role and produce an additional source of stress.

Outside of the family context it is found that individuals who engage in high levels of affectionate communication enjoy more social involvement [14]. Affectionate behavior such as laughter, smiling, praise, and excited verbal statements have been shown to elicit positive peer reactions in children [15]. Newcomb et al. [16] performed a meta-analysis of studies on peer relationships and found that popular children frequently exhibited prosocial behaviors (including affectionate actions) and made positive comments about others in comparison with children who were unpopular with peers. These findings suggest that affectionate communication may be a vital means through which children initiate interactions and form social relationships.

There are several hypotheses that can potentially explain the difficulties with affectionate behavior that individuals with AS experience. First, many children with AS experience heightened sensory perception [17], and sensitivity to touch has been found to occur in over 50% of children with AS [18]. It has been observed that some children with AS find gestures of affection such as a hug or kiss aversive, as the sensory experience is unpleasant [1, 19, 20]. Secondly, many individuals with AS have immature Theory of Mind skills and have difficulty recognising and understanding the thoughts, beliefs, desires, and intentions of others [21]. Therefore, it may not occur to a child with AS that another person is distressed and would appreciate physical comfort or verbal reassurance to help restore their emotional state. Thirdly, as well as difficulties recognising emotions in others and themselves [22], individuals with AS experience difficulties reading social cues within a social interaction. Poor interpretation of cues, such as the meaning of a facial expression, a gesture, or tone of voice, may limit expression of appropriate affectionate behavior [23]. A fourth explanation is that children with AS may simply lack insight as to what appropriate affection entails. For instance, an individual with AS may show that he/she cares by carrying out a practical deed (e.g., handing a distressed person a tissue), discussing his/her own special interest (would make them feel better), or leaving the sad person alone to self-soothe rather than displaying conventional forms of affection such as hugging. If this is the case, then it is possible to teach what is appropriate in different situations [1]. 

There is a small body of literature that has shown cognitive behavior therapy (CBT) to be effective in managing problems faced by young people with AS [24]. Randomized controlled trials have evaluated the efficacy of CBT interventions in this population for anxiety [25–28], for anger [29], and for social and emotional understanding [7]. Each of these trials has incorporated modifications to accommodate the cognitive profile of autism spectrum disorders.

The current study is a pilot trial of a 5-week intervention for children with AS that aims to increase understanding and appropriate use of affectionate behavior in a family setting. The intervention has been tailored for children with AS and uses strategies previously found to be effective. 

## 2. Intervention

The program has five 2-hour sessions for children that are held in small groups while the parents participate in a parallel parent session in a large group. In Session 1 the groups participate in “getting to know you activities” and focus on experiences and people that they like. They are introduced to the concrete concept of placing items on a visual scale and applying ratings (0–100) using post-it stickers. The session moves to identifying ways that we can tell if someone likes or loves us, again using visual strategies and then to why we express feelings of liking or loving people. The child is asked to complete some project work for the next session with the help of a parent.

Session 2 reviews the project work and moves on to constructing a Social Story [30] about how liking or loving someone can affect feelings and thoughts. Some time is spent constructing a schematic of the different people in each child's environment and how we may do and say different things to each one to show that we like them. There are then roleplays to practice this in the groups. The child is asked to practice showing affection to a parent or sibling in the following week and to report back to the group.

Session 3 reviews and demonstrates the practice done by each child in the week. The session then moves on to the concept of compliments and includes both discussion and roleplay around this for a variety of people. There is also a focus on receiving compliments and practice agreed for the home project.

In Sessions 4 and 5 the children continue to discuss their understanding of affection and to roleplay the various things that they can do and say in order to show a range of people that they like or love them. At the end of Session 5 there is a review of what they feel has been learned. The sessions are able to be tailored to address the specific concerns raised by each family and so different areas can be emphasized for individual children.

## 3. Method

### 3.1. Participants

Twenty-three children were recruited to participate in the study via media outreach through local newspapers and schools. One child with nonverbal autism was excluded from the trial, and two participants withdrew before the trial commenced. The final sample included twenty-one children (18 boys and 3 girls) aged 7 to 12 years with a mean age of 9.91 (SD = 1.56). Nineteen children had a primary diagnosis of AS, and two children had a primary diagnosis of high functioning autism (HFA). Participants resided in Brisbane, Australia. The Ethics Committee of the School of Psychology, University of Queensland, approved the study.

 Inclusion criteria for the study required that each child have a diagnosis of AS or HFA as confirmed by a pediatrician or a clinical psychologist as well as meeting criteria on the Asperger Syndrome Diagnostic Interview (ASDI; [31]) and an IQ score of 79 or higher on the Wechsler Abbreviated Scale of Intelligence (WASI; [32]). Difficulties with affectionate behavior were established based on parent report at the intake interview.

### 3.2. Procedures

As parents expressed an interest in participating in the program, they provided informed consent and were allocated a time for both parent and child to complete the interview and questionnaires. Children were allocated to small groups based on age and gender. There were three children in each group with two therapists to run the group. The parent group was held at the same time, and all parents were in one large group with two therapists. Each of the five 2-hour sessions was held on a Saturday. Measures were taken again immediately after program and at 3-month followup when families returned to attend an information session and receive certificates.

### 3.3. Measures

#### 3.3.1. Clinical Interview and General Affection Questions

A brief standardized clinical interview was administered to all parents prior to the intervention. The interview included questions pertaining to the child's demographics, diagnoses, current medications, and problems with affectionate behavior. The ASDI was also administered as part of the intake procedure.

#### 3.3.2. Asperger Syndrome Diagnostic Interview (ASDI)

The ASDI [31] is a 20-item structured interview designed to capture the degree to which children display traits associated with AS. The items assess the symptoms included in the diagnostic criteria for AS developed by I. C. Gillberg and C. Gillberg [33] including social impairment, narrow interest, compulsive need for routines and interests, speech and language peculiarities, nonverbal communication problems, and motor clumsiness. Each item is rated as 0 or 1, with 0 = criterion not met and 1 = criterion is met. Gillberg et al. [31] reported excellent interrater reliability (kappa =  .91) and intrarater reliability (kappa =  .92).

#### 3.3.3. Wechsler Abbreviated Scale of Intelligence (WASI)

The WASI [32] is a standardised individually administered test of cognitive functioning in individuals aged 6–89 years. It is an abbreviated measure of intelligence and consists of four subtests: Vocabulary, Block Design, Similarities, and Matrix Reasoning. The examiner also has the option of administering the two-subtest format (i.e., Vocabulary and Matrix Reasoning), and this was used in the current study. The WASI gives a global measure of intellectual ability.

#### 3.3.4. Affection for Others Questionnaire (AOQ)

The AOQ [34] is a newly developed measure that aims to assess a child's capacity to engage in affectionate behavior with “others” (i.e., people outside of the child's immediate family such as teachers, classmates, family friends, and professionals). The AOQ was designed with five subscales: Giving Verbal Affection to Others, Giving Physical Affection to Others, Receiving Verbal Affection from Others, Receiving Physical Affection from Others, and Communicating Empathy to Others. The Cronbach's alpha coefficient for the subscales of the AOQ ranged from *α* = .85 to *α* = .94 in the present study, suggesting high internal consistency. Each subscale includes 4 questions making a total of 20 questions. Each item has two parts. The first asks parents to rate whether their child was able to complete each of the affectionate gestures appropriately (ranging from 1, “Never Appropriate”, through to 7, “Always Appropriate”). The second part asked parents to provide a description of the amount that their child displayed this affectionate gesture (with responses ranging from 1, “Not Enough”, to 7, “Too Much”). An “Appropriateness” score and an “Amount” score were computed for the Giving Affection, Receiving Affection, and Communicating Empathy subscales, and a “Total Appropriateness” score and a “Total Amount” score were calculated by adding the totals for each subscale. 

#### 3.3.5. Affection for You Questionnaire (AYQ)

The AYQ [34] is another newly developed measure that aims to assess a child's ability to engage in affectionate interactions with their parents. The AYQ was developed with five subscales: Giving Verbal Affection to You, Giving Physical Affection to You, Receiving Verbal Affection from You, Receiving Physical Affection from You, and Communicating Empathy to You. Alpha coefficients for the subscales ranged from *α* = .90 to *α* = .95 in the current sample. The number of questions making up each subscale varies from 3 to 5 items per subscale, yielding a total of 19 questions. There were two parts to each question. The first asked parents to rate how often the child completed the affectionate gesture (ranging from 1, “Never”, through to 7, “Twice a day or more”). The second part asked the parent to provide a description of the amount (ranging from 1, “Not Enough,” to 7, “Too Much”). The first part of the question was only intended for qualitative purposes, and therefore no total score was calculated for these items. As with the AOQ, an “Amount” score was computed for each subscale, and a “Total Amount” score was calculated by adding the totals for each subscale. 

#### 3.3.6. General Affection Questionnaire (GAQ)

The GAQ [34] aims to assess a child's general difficulty with affectionate behavior. The GAQ comprises two subscales: Excessive Affection and Inadequate Affection. The GAQ has 12 statements that assess the amount of affection in which the child engages (e.g., “He/she shows a lack of affection”), the appropriateness of the affection a child expresses (e.g., “He/she uses inappropriate expressions of affection”), the impact that difficulties with affection has on various areas of the child's life (e.g., “He/she has difficulties with affection that cause problems with his/her siblings”), and the child's knowledge of affection (e.g., “I have had to spend time teaching him/her about affection”). The scale ranges from 1, “Strongly Disagree,” to 7, “Strongly Agree”. The sum of all 12 items yields a “Total Difficulty with Affection” score. Alpha coefficients were adequate (*α* = .75 and  .87) in the current sample. 

#### 3.3.7. Touch Inventory for Elementary-School-Aged Children (TI)

The TI [35] is a 26-item parent report scale designed to measure a child's tactile sensitivity. Each item requires the participant to indicate the level of their child's reaction to various forms of touch (e.g., “does it bother your child to be hugged or held?” or “does it bother your child to have their face touched?”). The TI has a high test-retest reliability with a seven-day interval (*r* = .91, *P* < .01) [36]. The TI has also demonstrated discriminant validity with an ability to differentiate between children identified as being tactile defensive and nontactile defensive with an 85% correct classification rate (Wilk's Λ = .58, *df* = 26, *P* = .007) [37]. The scale showed good reliability in the present study with *α* = .87. 

#### 3.3.8. Social Skills Questionnaire-Parent(s) (SSQ-P)

The *SSQ-P *[38] is a 30-item parent report questionnaire designed to assess a child's social skills in the four weeks prior to completion of the measure. Parents are asked to rate the accuracy of a series of statements (ranging from “Not true” to “Mostly true”). The questionnaire showed good reliability in the present study, *α* = .93.

#### 3.3.9. Social Competence with Peers Questionnaire-Parent(s) (SCPQ-P)

The *SCPQ-P* [38] consists of 9 statements regarding a child's social competence with peers in the past four weeks. Parents are asked to rate the extent to which each of the statements is true (ranging from “Not true” to “Mostly true”). The measure correlates significantly with teacher and child ratings of competence with peers and with the *SSQ-P*. The scale showed good reliability with the current sample *α* = .90. 

#### 3.3.10. Walk in the Forest Test (WFT)

The WFT is a measure specifically designed by Attwood [39] for the current study to assess a child's understanding of affection. The measure consists of a hypothetical scenario that describes an interaction between the child and an alien in the forest. During the interaction, the alien asks the child why humans are affectionate with each other. The child is then prompted to generate possible reasons that people express affection. Administration is standardised, with the scenario read aloud to the child by the examiner, and the child's responses recorded and rated for appropriateness. The WFT is scored by allocating one point for each appropriate response. Two independent examiners were used to evaluate interrater reliability, which was 98%. 

## 4. Results

Analyses were conducted using the statistical computer program PASW Statistics for Windows Version 17.0. Four missing values were identified and replaced using mean substitution. Twelve of the participants attended all five sessions, and one child withdrew from the study after attending session one. The data for this child was handled using an intention-to-treat procedure. Family-wise Bonferroni correction was conducted to limit the likelihood of Type 1 errors in the univariate analyses [40]. The significance levels determined after the Bonferroni adjustment were *P* < .006 for all the mean comparisons. The significance levels used for the correlations were *P* < .05. 

### 4.1. Correlations

The correlations discussed in this section are presented in Table 1. The total scores on the GAQ and the total scores on the SCPQ-P were significantly inversely correlated. The total scores on the GAQ and the total scores on the SSQ-P were significantly negatively correlated. These findings indicate that, as general difficulty with affection increases, social ability decreases. The correlation between the total scores on the AOQ and the total scores on the SSQ-P was significant. In addition, the correlation between total scores on the AOQ and the total scores on the SCPQ-P was significant. These results suggest that as the appropriateness of affection increases, then so does social ability. 

### 4.2. Intervention Effects

#### 4.2.1. General Problems with Affection

The child's general difficulty with affection was measured via parent report using the GAQ. A series of within-subjects, repeated measures ANOVAs was conducted to compare parent reports of child affection difficulties across time (pre-, post-, and followup). Results from the GAQ showed that the mean total scores on the GAQ were not significantly different from pre- to postintervention, and this result was maintained at followup.

#### 4.2.2. Appropriateness of Affection with Others

Appropriate expression of affectionate behavior towards “others” (i.e., people other than immediate family) was measured by parent report on the AOQ. The “Total Appropriateness” score was used to measure this construct. Within-subjects repeated measures ANOVAs were conducted to compare parent reports of the children's ability to engage in appropriate affection across time (pre-, post-, and followup). Analyses were conducted for the total scale and for the three subscales of the AOQ, “Giving Affection to Others,” “Receiving Affection from Others,” and “Communicating Empathy to Others.” 

Results from the AOQ showed that the mean total scores on the AOQ were significantly different from pre- to postintervention, *F*(1, 20) = 15.403, *P* < .001, partial *η* ^2^ = .435, indicating that parents reported an increase in the appropriateness of the affectionate interactions between children and “others” at postintervention, and this finding was maintained at followup. 

Results from the “Giving Affection” subscale of the AOQ showed that mean scores on the Giving Affection subscale of the AOQ were significantly different from pre- to postintervention, *F*(1, 20) = 15.254, *P* < .001, partial *η* ^2^ = .433, such that parents reported an increase in the appropriateness of the children's ability to give affection to others after intervention, and this was maintained at followup.

Results from the “Receiving Affection” subscale of the AOQ showed that mean scores on the Receiving Affection subscale of the AOQ were not significantly different from pre- to postintervention, and this was maintained at followup.

Results from the “Communicating Empathy” subscale of the AOQ showed that mean scores on the Communicating Empathy subscale of the AOQ were significantly different from pre- to postintervention, *F*(1, 20) = 10.057, *P* < .005, partial *η* ^2^ = .335, with parents reporting an increase in the appropriateness of the children's ability to communicate empathy to others after intervention. This result was maintained at followup. Means and standard deviations are provided in Table 2.

#### 4.2.3. Understanding of the Purpose of Affection

The child's understanding of the purpose of affection was assessed via child report using the “A Walk in the Forest Test.” A within-subjects repeated measures ANOVA was conducted to compare children's understanding of the function of affection across time. Results from the WFT showed that the mean scores on the WFT were significantly different from pre- to postintervention, *F*(1, 20) = 21.929, *P* < 0.001, partial *η* ^2^ = .523, indicating that the children's understanding increased after intervention, and this was maintained at followup. 

#### 4.2.4. Amount of Affection Expressed

Parents' perceptions of the amount of affection their child expressed to others (i.e., people outside their immediate family) and to them as parents were assessed by means of the AOQ and AYQ. The Total Amount score from each scale was summed to measure this construct. In this section, the number of children showing improvement was examined, in order to give a more meaningful description of the results in terms of clinical outcomes. No statistical analyses were applied to this data as the scale on the AOQ and AYQ where parents rate their perception of the affectionate behavior of their child is not linear; that is, increases as well as decreases on this rating scale can suggest improvement in the child's appropriate demonstration of affectionate behavior as long as the child's score moved towards the middle of the scale. 

Participants were divided into three groups based on their baseline Total Amount scores derived from the AOQ and AYQ. The participants with baseline Total Amount scores of 59 and below on the AOQ and of 57 and below on the AYQ were categorized as the “Low Affection” group. The participants with baseline scores between 59 and 100 on the AOQ and between 57 and 95 on the AYQ were labeled as the “Adequate Affection” group. Participants with baseline scores of 101 and over on the AOQ and of 96 and over on the AYQ were regarded as the “High Affection” group. The results are displayed in Table 3. 

Total Amount scores on the AOQ indicate that there was a substantial increase from pre- to postintervention in the number of children reported by parents to express more adequate levels of affection to others with eight children (38.1%) moving from the low affection to the adequate affection category, and this was maintained at followup. Total Amount scores on the AYQ at pre- and postintervention also indicate that there was a marked increase in the number of children perceived by their parents to express adequate levels of affection to them as parents. Nine children (42.86%) moved from the low affection to the adequate affection group, and one child moved from the high affection group with results maintained at followup. 

#### 4.2.5. Qualitative Findings

At postintervention, parents were asked to complete a brief questionnaire regarding their experience of the program. The majority of parents reported that they observed improvements in their child's affectionate behavior after completing the program. These improvements included an increased understanding of affectionate behavior and its importance, an increased awareness of affectionate behavior in self and others, higher levels of giving verbal (e.g., compliments) and nonverbal (e.g., hugs and kisses) affection, more tolerance in terms of receiving verbal and nonverbal affection, and an increased capacity to engage in appropriate affectionate behaviors. Some parents stated that the program provided a good foundation on which to build and refine their child's skills in affectionate behavior. These qualitative findings are consistent with the empirical results. 

## 5. Discussion

Children with Asperger syndrome have a propensity to be inappropriate in their expression of affectionate behavior [1]. The results from this pilot study indicated a significant increase in the overall appropriateness of children's affectionate behavior towards others (i.e., those people outside their immediate family) reported by parents at postintervention and followup. In particular, there was a significant improvement in the appropriateness of affection given and the empathy communicated to others. However, there was no significant improvement in the appropriateness of children's responses to the affection they received from others. A possible explanation for this finding is that, although some work was done on responding appropriately to affection initiated by others, the program predominantly focussed on initiating appropriate affection. It is also the case that children with AS do not like the unexpected and whereas they can control self-initiated affectionate behaviors they are unable to control those behaviors initiated by others. This may also be associated with tactile sensitivity issues.

A marked increase was reported in the number of children that changed categories to be described by parents as engaging in more adequate levels of affectionate behavior, both toward parents and toward individuals outside the immediate family. Although it is unclear whether these improvements are statistically significant, it certainly appears that the “Exploring Feelings: Affection” program is effective in increasing affectionate behavior in those children who exhibit unusually low levels of affection. These results are consistent with the findings of previous studies that demonstrated that children with an ASD can learn to be more affectionate (e.g., [11, 41, 42]). More specifically, these outcomes support the findings of Twardosz et al. [42] who found that discussing and practising affectionate behavior can increase expressions of affection by children with autistic features. The program was also effective in reducing the amount of affection expressed by the one high affection communicator identified in the study.

Children with AS may have an immature understanding of why affectionate behavior is important [1]. The results suggested that there was a significant improvement in child understanding of the purpose of affection at postintervention and followup. This finding suggests that the children not only made changes on a behavioral level but also at a cognitive level. This is important as individuals with AS are typically very logical and if they perceive the usefulness of engaging in certain behaviors they will be more likely to modify their behavior [1]. 

### 5.1. Qualitative Findings

The qualitative data collected from the study, congruent with empirical findings, provide powerful testimony to the program's application for creating positive change within households. Parents observed improvements in their child's understanding and consciousness of affectionate behavior, displays of verbal and nonverbal affection, tolerance of receiving verbal and nonverbal affection, and in the appropriateness of affectionate behaviors. Some parents believed that the program would provide a good foundation for assisting their child to develop further skills in affectionate behavior in the future. This potential is apparent in those cases where improved scores on the “A Walk in the Forest” test were maintained at followup, implying a retained insight into the purpose and importance of affection after completing the program and 3 months after the intervention. 

### 5.2. Limitations and Future Directions

There are some limitations that need to be taken into consideration when interpreting the results of this research. First, the outcomes were primarily evaluated by means of parent-report questionnaires. Therefore, as with many studies that rely on the report of parents, the data is subjective and may be open to bias. Also, some parents reported that they had been diagnosed with AS, which may have impacted on their ability to produce reliable data; that is, individuals with AS can be very black and white in their thinking [1] and, therefore, when answering a questionnaire, may have a tendency to provide extreme responses that may over- or underestimate their child's actual abilities. It may therefore be beneficial to employ observations of parent-child interactions in future research to provide objective findings. The positive change in the parents' perception of their children's affectionate behavior is nonetheless an important outcome achieved through the current intervention. 

## Figures and Tables

**Table 1:** Correlations between measures of affection and other variables.

	GAQ	AOQ	Walk in the Forest
Touch Inventory	.384	−.258	.175
Social Skills (SSQ-P)	−.759**	.831**	−.104
Social Competence (SCPQ-P)	−.633**	.710**	.213

GAQ: General Affection Questionnaire; AOQ: Affection for Others Questionnaire; *N* = 21; **P < .001.

**Table 2:** Means scores on the AOQ by testing time.

Measure	Means					
	Pre-		Post-		Followup	
	M	SD	M	SD	M	SD
AOQ total	68.67	(25.46)	84.91**	(17.31)	83.43	(16.63)
AOQ—Giving Affection	25.67	(9.33)	33.00**	(7.18)	31.86	(7.58)
AOQ—Receiving Affection	29.14	(12.76)	34.43	(7.16)	33.95	(6.40)
AOQ—Comm. Empathy	13.86	(5.84)	17.48*	(4.52)	17.62	(4.30)

*Significantly higher than preintervention score at *P* < .006.

**Significantly higher than preintervention score at *P* < .001.

**Table 3:** Number and percentage (parentheses) of participants in the low-affection, adequate affection, and high-affection groups at pre-, post-, and followup.

Group	Time	AOQ	AYQ
Low affection	Pre	14 (66.67%)	9 (42.86%)
	Post	6 (28.57%)	1 (4.76%)
	Followup	6 (28.57%)	3 (14.29%)

Adequate affection	Pre	7 (33.33%)	11 (52.38%)
	Post	15 (71.43%)	20 (95.24%)
	Followup	15 (71.43%)	18 (85.71%)

High affection	Pre	0	1 (4.76%)
	Post	0	0
	Followup	0	0
